# Evaluation of Fungal Growth on Olive-Mill Wastewaters Treated at High Temperature and by High-Pressure Homogenization

**DOI:** 10.3389/fmicb.2017.02515

**Published:** 2017-12-14

**Authors:** Francesca Cibelli, Antonio Bevilacqua, Maria L. Raimondo, Daniela Campaniello, Antonia Carlucci, Claudio Ciccarone, Milena Sinigaglia, Maria R. Corbo

**Affiliations:** Department of the Science of Agriculture, Food and Environment, University of Foggia, Foggia, Italy

**Keywords:** fungi, high-pressure of homogenization, thermal treatment, olive mill wastewater, antifungal activity

## Abstract

Reuse of olive mill wastewaters (OMWWs) in agriculture represents a significant challenge for health and safety of our planet. Phytotoxic compounds in OMWW generally prohibit use of untreated OMWWs for agricultural irrigation or direct discharge into surface waters. However, pretreated OMWW can have positive effects on chemical and microbiological soil characteristics, to fight against fungal soil-borne pathogens. Low amounts of OMWW following thermal (TT-OMWW) and high-pressure homogenization (HPH-OMWW) pretreatments counteracted growth of some of 12 soil-borne and/or pathogenic fungi examined. With fungal growth measured as standardized change in time to half maximum colony diameter, Δτ, overall, HPH-OMWW showed increased bioactivity, as increased mean Δτ from 3.0 to 4.8 days. Principal component analysis highlighted two fungal groups: *Colletotrichum gloeosporioides, Alternaria alternata, Sclerotium rolfsii*, and *Rosellinia necatrix*, with growth strongly inhibited by the treated OMWWs; and *Aspergillus ochraceus* and *Phaeoacremonium parasiticum*, with stimulated growth by the treated OMWWs. As a non-thermal treatment, HPH-OMWW generally shows improved positive effects, which potentially arise from preservation of the phenols.

## Introduction

Phenols, long chain fatty acids and other phytotoxic and inhibitory compounds are the major constituents of olive-mill wastewater (OMWW); however, the exact composition of OMWW is greatly variable and relies upon some factors like region of production process, milling conditions (Niaounakis and Halvadakis, 2006).

Phenols and other compounds limit the use of OMWW in agriculture, as they could affect the chemical and physical traits of soil (porosity and pH) and the germination of seeds (Marrara et al., 2002; El Hadrami et al., 2004; Mekki et al., 2006). Moreover, OMWW, when discharged directly into surface waters, can discolor streams and rivers (Niaounakis and Halvadakis, 2006).

However, OMWW can be also a valuable source for many purposes (production of fertilizers, recovery of antioxidants, and production of biopolymers, biogas and animal feed) (Morillo et al., 2009). In a recent study, Disciglio et al. (2015) investigated the effects of irrigation with treated agro-industrial wastewater on soil and fungal populations. Compared to groundwater, wastewater had significantly higher electrical conductivity, total suspended solids, sodium, calcium, magnesium, potassium and sodium adsorption ratios, chemical oxygen demand, biological oxygen demand over 5 days, ammonium–nitrogen, and phenol, bicarbonate, phosphate, sulfate and chloride contents. Most of these characteristics were significantly higher also in the wastewater-irrigated soil. During the tomato cultivation, there were significant shifts in the composition of the soil microbial community. Saprophytic species increased in the wastewater-treated soil, while plant pathogens, such as *Fusarium oxysporum*, progressively decreased.

Other studies have reported that when the high content of organic matter in wastewater (compared to fresh water) decomposes in the soil, this can increase the population of saprophytic fungi. Some of these can act as antagonists of soil-borne plant pathogens, such as *Trichoderma* spp., *Fusarium* spp., and *Aspergillus* spp. (Manici et al., 2004). Thus, the use of treated agro-industrial wastewater can provide great benefits to agriculture as well as to environmental recovery, because the use of intensive agricultural systems can lead to a major build-up of soil-borne plant pathogens, as reported by Garibaldi and Gullino (1995). Lops et al. (2008) showed that milling waste could be effective in the control of some fungal diseases and for plant growth. This was believed to arise through the polyphenolic compounds that it contains, which can be useful against fungal soil-borne pathogens that cause root rot.

The use of OMWW in agriculture also pose the problem of a preliminary treatment due to the high level of the naturally occurring microbiota (Sinigaglia et al., 2010). Thermal treatment could be a possible approach, but it could also result in some undesirable physico-chemical changes, like phenol degradation. Thus, non-thermal technologies have been proposed to provide cold pasteurization, which would reduce this heat damage. High-pressure homogenization (HPH) and high hydrostatic pressure are promising non-thermal technologies that are particularly suitable for continuous production of liquid foods while limiting thermal damage (Xi et al., 2009; Suárez-Jacobo et al., 2010; Calligaris et al., 2012; Varela-Santos et al., 2012; Velázquez-Estrada et al., 2013; Amador-Espejo et al., 2014; Yu et al., 2014; Wibowo et al., 2015).

The focus of this study was on OMWW as a tool to control and/or inhibit soil-borne fungi in agriculture. The OMWW was initially treated and stabilized to reduce the naturally occurring microbiota to acceptable levels. The two approaches used here were conventional thermal treatment (TT-OMWW) and HPH (HPH-OMWW), to determine whether these different approaches act on bioactivities in different ways.

## Materials and methods

### Olive-mill wastewater collection and testing

The OMWW used in this study was collected from a local olive mill in the province of Foggia (southern Italy) that operated using both continuous and traditional oil extraction methods. Thus, this OMWW was a mixture of the wastewaters from both of these extraction methods. It was subjected to pH and phenolics determinations using the methods described by Bray and Thrope (1954). Moreover, proteins and reducing sugar were assessed as respectively reported by Bradford (1976) and De Clerk (1963, Fehling method).

### Olive-mill wastewater treatments

In order to obtain TT-OMWW used in this study, OMWW was diluted in agar and sterilized at 121°C for 20 min.

In order to obtain the HPH-OMWW, OMWW was filtered through Miracloth paper (Calbiochem, Germany), and processed using a high-pressure homogenizer (PANDA 2K; Niro Soavi s.p.a., Parma, Italy), at 150 MPa. The homogenizer was a two-valve device. The first valve (a ball valve, indicated by the manufacturer as the “main valve”) worked at a maximum pressure of 150 MPa and was used for the homogenization. The second valve was designed for emulsion preparation and micronization treatment, and worked at a maximum pressure of 15 MPa. In the present study, the HPH was performed only through the main valve. Before each treatment, the equipment was cleaned with sterilized distilled water at 70°C, and cooled with sterilized distilled water to obtain an exit temperature of the samples of < 35°C.

### Fungi

Twelve reference fungal species from a fungal pool assayed on different OMWWs in a previous study (Bevilacqua et al., 2017) were used to examine their growth on TT-OMWW and HPH-OMWW in the present study. Information about these fungal species is given in Table 1, which were: *Lasiodiplodia theobromae* (Pat.) Griffon & Maubl., *Colletotrichum gloeosporioides* (Penz.) Penz. & Sacc., *F. oxysporum* E. F. Sm. & Swingle, *Phaeoacremonium parasiticum* (Ajello, Georg & C. J. K. Wang) W. Gams, Crous & M. J. Wingf., *Penicillium italicum* Wehmer, *Aspergillus niger* Tiegh., *Aspergillus ochraceus* G. Wilh., *Alternaria alternata* (Fr.) Keissl., *Trichoderma* sp. Pers., *Sclerotium rolfsii* Sacc., *Rosellinia necatrix* Berl. ex Prill., and *Diaporthe amygdali* Udayanga, Crous & K. D. Hyde.

**Table 1:** Details of the fungal species used in this study.

**Fungal species**	**Host**	**Locality**	**Identification tools**	**Reference or collection**	**Abbreviation**
*Alternaria alternata*	*Olea europea*	Cerignola, Foggia, Italy	MOR	Carlucci et al., 2015b	Aal
*Aspergillus niger*	Oil-mill wastewater	Cerignola, Foggia, Italy	MOR	Dept. SAFE	Ang
*Aspergillus ochraceus*	Soil	Stornarella, Foggia, Italy	MOR	Dept. SAFE	Aoc
*Colletotrichum gloeosporioides*	*Olea europea*	Matino, Lecce, Italy	MOR	Dept. SAFE	Cgd
*Diaporthe amygdali*	*Prunus dulcis*	Matino, Lecce, Italy	MC	Dept. SAFE	Dag
*Fusarium oxysporum*	*Olea europea*	Presicce, Lecce, Italy	MC	Dept. SAFE	Fx
*Lasiodiplodia theobromae*	*Vitis vinifera*	Cerignola, Foggia, Italy	MC	Carlucci et al., 2015a	Bot
*Penicillium italicum*	Soil	Stornarella, Italy	MOR	Dept. SAFE	Pit
*Phaeoacremonium parasiticum*	*Olea europea*	Cerignola, Foggia, Italy	MC	Carlucci et al., 2015b	Pmp
*Rosellinia necatrix*	*Olea europea*	Cerignola, Foggia, Italy	MOR	Dept. SAFE	Rnx
*Sclerotium rolfsii*	*Solanum lycopersicum*	Stornarella, Foggia, Italy	MOR	Dept. SAFE	Sri
*Trichoderma sp*.	Soil	Stornarella, Foggia, Italy	MOR	Dept. SAFE	Trc

*MC, molecular characterization; MOR, morphological and microscopic characterization; Dept. SAFE, Department of the Science of Agriculture, Food and Environment, University of Foggia, Foggia, Italy*.

### Growth rates on the treated olive-mill wastewater minimal media

Fungal growth was initially assessed in a minimal culture medium using TT-OMWW as the only source of nutrition. The TT-OMWW was diluted in distilled water containing 15 g L^−1^ agar (Agar Technical n.3; Oxoid Ltd., UK) to three final concentrations: 5, 10, and 15%. These media were sterilized at 121°C for 20 min (as the thermal treatment), and then poured into sterile 55-mm-diameter Petri dishes. The fungi were aseptically inoculated using 2-mm mycelium plugs cut from the colony margins of 7–10-day-old cultures of the target fungi in Petri dishes, with each assay run in triplicate.

Similarly, the fungal growth was assessed in a minimal culture medium using HPH-OMWW as the only source of nutrition. The HPH-OMWW was also diluted in distilled water containing 15 g L^−1^ agar (Agar Technical n.3; Oxoid Ltd., UK) to the same three final concentrations: 5, 10, and 15%. These media were poured directly into sterile 55-mm-diameter Petri dishes. The fungi were inoculated as described above, with each assay run in triplicate.

The media inoculated with TT-OMWW and HPH-OMWW were then incubated at 23 ± 2°C in the dark. The control cultures that used PDA as the positive control were prepared and incubated in parallel.

The colony diameters were measured and compared with those of the PDA control plates at intervals of 48 h over 21 days, to determine the radial growth of these fungi under these minimal growth conditions with TT-OMWW and HPH-OMWW as the only sources of nutrition (Carlucci et al., 2012).

### Statistical analysis and modeling

The experiments were performed in triplicate. Fungal growth was modeled using a logistic equation as modified by Dantigny et al. (2011), and given in Equation (1):

$$\begin{array}{l} D=\frac{D_{\max}}{1+\exp\left[k\left(\tau -t\right)\right]} \end{array}$$

where D is the diameter of the fungal colony at the chosen time, *D*_max_ is the maximum diameter of the fungal colony (here set as 55 mm; i.e., the diameter of the plates), *k* is the rate of fungal growth (mm day^−1^), τ is the time to attain half *D*_max_ (days), and t is the chosen time of the analysis (days).

The differences among the fitting parameters from OMWW at different concentrations were assessed through one-way ANOVA; the test of Tukey was used as the *post-hoc* test (*P* < 0.05) (Statistica for Windows).

Data fitting was performed using the Statistica for Windows software (version 12.0; Statsoft, Tulsa, OK, USA), with a least-squares approach. After this primary modeling step, secondary modeling was performed. Before this step, τ was standardized as given in Equation (2):

$$\begin{array}{l} \tau =\tau_{OMW}-\tau_{c} \end{array}$$

where τ_*OMW*_ and τ_*c*_ are the τ values from the media supplemented with TT-OMWW or HPH-OMWW and from the control medium with PDA, respectively. A positive Δτ indicates inhibition of fungal growth due to the TT-OMWW and HPH-OMWW, whereas a negative Δτ indicates that the fungal growth was promoted.

This Δτ was then used as the dependent variable to run multi-factorial analysis of variance (ANOVA). The categorical predictors (i.e., independent variables) were the treatment (TT-OMWW, HPH-OMWW) (T), the amount of these OMWWs additions (5, 10, 15%) (A), and the fungal species assayed (F). Such multi-factorial ANOVA offers two kinds of outputs:

- A table of standardized effects that shows the Fisher-test factors and significance of the individual (i.e., F, A, T) and interactive (i.e., F^*^A, T^*^A, F^*^T, F^*^T^*^A) effects of the predictors.
- Hypothesis decomposition graphs that show the correlation of each predictor or interactive effect vs. Δτ.

As the final step, Principal Component Analysis (Statistica for Windows) was run using the Δτ from the TT-OMWW and HPH-OMWW analysis, with the media supplemented with 5–15% of each treated OMWW.

## Results

The equation of Dantigny et al. (2011) satisfactorily fitted all of these datasets, with regression coefficients that ranged from 0.937 to 0.999.

Table 2 gives the fitting parameters from the equation of Dantigny et al. (2011) for the 12 fungi tested here. These fungi showed different trends, mainly on the basis of their saprophytic and parasitic roles. A first group comprised *L. theobromae, Pm. parasiticum*, and *D. amygdali*, which are trunk disease pathogens of several fruit trees (Mostert et al., 2006; Diogo et al., 2010; Carlucci et al., 2015a,b). On the 15% OMWW-supplemented agars, these three fungi generally experienced an increase in τ (TT-OMWW, 3.26 ± 0.05 days to 14.26 ± 0.71 days; HPH-OMWW, 5.07 ± 0.25 days to 14.04 ± 0.06 days), with some exceptions: *D. amygdali* was not affected by TT-OMWW, while HPH-OMWW promoted increased τ in *Pm. parasiticum* only at 15%, but it showed τ level lower than positive control in 5–10% HPH-OMWW and TT-OMWW. *C. gloeosporioides* and *A. alternata* are the causal agents of anthracnose and black spot disease on olive drupes (Moral et al., 2008; Talhinhas et al., 2015), and these fungi were affected by both TT-OMWW and HPH-OMWW, where τ ranged from 3.03 ± 0.03 days (5%) to 16.32 ± 0.04 days (15%), and from 6.81 ± 0.12 days (5%) to 22.0 ± 0.10 days (15%), respectively. *F. oxysporum* is a soil-borne fungus, and it was not affected by TT-OMWW at 5%. Similarly, the growth of *Trichoderma* sp. was not influenced by TT-OMWW, while on HPH-OMWW, τ increased from 2.93 ± 0.17 days (5%) to 15.95 ± 0.01 days (15%). *S. rolfsii* and *R. necatrix* are severe soilborne fungal pathogens of horticultural crops and fruit trees, respectively, as reported by Lops et al. (2008) and Carlucci et al. (2013), and their growth was inhibited by 10 and 15% TT-OMWW.

**Table 2:** Fungi grown on the laboratory media supplemented with either thermal-treated or high-pressure homogenized OMWW.

**Fungal species**	**Time to attain half maximum diameter on supplemented agar (τ, days)**						
	**Potato Dextrose**	**Thermal treated OMWW agar (%)**			**High-pressure homogenized OMWW agar (%)**		
	**Agar**	**5**	**10**	**15**	**5**	**10**	**15**
*Alternaria alternata*	2.07 ± 0.01A	3.03 ± 0.03B	6.02 ± 0.16C	9.05 ± 0.16E	6.81 ± 0.12C	7.76 ± 0.08D	15.97 ± 0.01F
*Aspergillus niger*	4.11 ± 0.14A	4.96 ± 0.29A, B	5.72 ± 0.27B	5.69 ± 0.27B	4.65 ± 0.21A	9.38 ± 0.22C	15.97 ± 0.01D
*Aspergillus ochraceus*	10.25 ± 0.29D	8.31 ± 0.21C	7.12 ± 0.28B	22.21 ± 0.60	5.46 ± 0.16A	9.85 ± 0.21D	15.89 ± 0.01E
*Colletotrichum gloeosporioides*	2.68 ± 0.10A	3.99 ± 0.12B	8.85 ± 0.16C	16.32 ± 0.40E	8.51 ± 0.20C	11.05 ± 0.22D	22.00 ± 0.10F
*Diaporthe amygdali*	6.49 ± 0.22B	3.65 ± 0.13A	4.19 ± 0.08A	6.47 ± 0.14B	7.73 ± 0.14C	10.11 ± 0.21D	14.03 ± 0.06E
*Fusarium oxysporum*	3.02 ± 0.07B	1.90 ± 0.01A	4.07 ± 0.14C	4.98 ± 0.15D	6.27 ± 0.24E	7.44 ± 0.07F	15.99 ± 0.10G
*Lasiodiplodia theobromae*	2.00 ± 0.01A	1.78 ± 0.0A3	2.03 ± 0.1A	3.26 ± 0.05B	5.07 ± 0.25C	8.06 ± 0.21D	9.99 ± 0.10E
*Penicillium italicum*	5.32 ± 0.38A	8.28 ± 0.12C	6.88 ± 0.14A, B	7.28 ± 0.15B	7.27 ± 0.12B	7.26 ± 0.11B	11.54 ± 0.10D
*Phaeoacremonium parasiticum*	10.78 ± 0.14C	6.96 ± 0.15A	8.87 ± 0.18B	14.26 ± 0.71D	9.14 ± 0.19B	9.85 ± 0.24B, C	14.04 ± 0.06D
*Rosellinia necatrix*	3.46 ± 0.02A	4.84 ± 0.14B	–	–	7.65 ± 0.16C	9.9 ± 0.02D	11.55 ± 0.10E
*Sclerotium rolfsii*	1.89 ± 0.01A	7.54 ± 0.03B	–^*^	–	7.05 ± 0.14B	7.51 ± 0.13B	11.55 ± 0.10C
*Trichoderma* sp.	1.81 ± 0.02A	1.87 ± 0.02A	2.05 ± 0.10A	2.14 ± 0.10A	2.93 ± 0.17B	6.30 ± 0.03C	15.95 ± 0.01D

*Data are means ± standard error. The letters indicate the significant differences in each row (one-way ANOVA and Tukey's test, P < 0.05)*.

* *No growth*.

A second group experienced different trends for τ, where this was lower than the PDA positive control. *A. ochraceus* was characterized by lower τ on the media containing 5% and 10% TT-OMWW and HPH-OMWW, and it was inhibited only with the 15% TT-OMWW and HPH-OMWW, as seen by the increased τ to 22.21 ± 0.60 days and 15.89 ± 0.01 days, respectively, as well as *Pm. parasiticum*. A similar situation was seen for *A. niger*, which used the phenolic compounds of TT-OMWW for its growth more so than HPH-OMWW.

Although Table 2 offers an interesting overview of the growth kinetics of these fungi with TT-OMWW and HPH-OMWW, the fitting parameters for these different fungal species could not be simply compared. Thus, the τ values were standardized to provide Δτ, in terms of their increases/decreases compared to the PDA positive control. Then, these Δτ were used to run multi-factorial ANOVA according to three different independent factors: the fungus (F), the percentage (amount) treated OMWWs added (A), and the OMWW treatments (T). Table 3 shows the statistical analysis and *F*-values for both of these individual and the interactive terms. These data indicate that Δτ was mainly affected by the amount of treated OMWWs added, followed by the fungal species, and finally by the OMWW treatments. For the interactive terms, the most significant effect was for the fungi versus OMWW treatment interaction (F^*^T).

**Table 3:** Multifactorial ANOVA run on the parameter Δτ (prolongation of τ with reference to the PDA positive control; days).

**Factor**	**Sum of squares**	**Degrees of freedom**	**Mean sum of squares**	**Fisher test**	***p***
Intercept	4478.16	1	4478.15	126769.3	0.00
F	2578.06	11	234.37	6634.5	0.00
T	175.70	1	175.70	4973.9	0.00
A	1931.22	2	965.61	27334.8	0.00
F*T	1263.93	11	114.90	3252.7	0.00
F*A	639.48	22	29.07	822.8	0.00
A*T	72.82	2	36.41	1030.6	0.00
F*A*T	691.42	22	31.43	889.7	0.00
Error	5.09	144	0.04		

*F, effect of fungal species (Table 1); A, effect of amount of treated OMWWs (5, 10, 15%); T, effect of OMWW treatments (TT-OMWW, HPH-OMWW)*.

**Figure 1:**
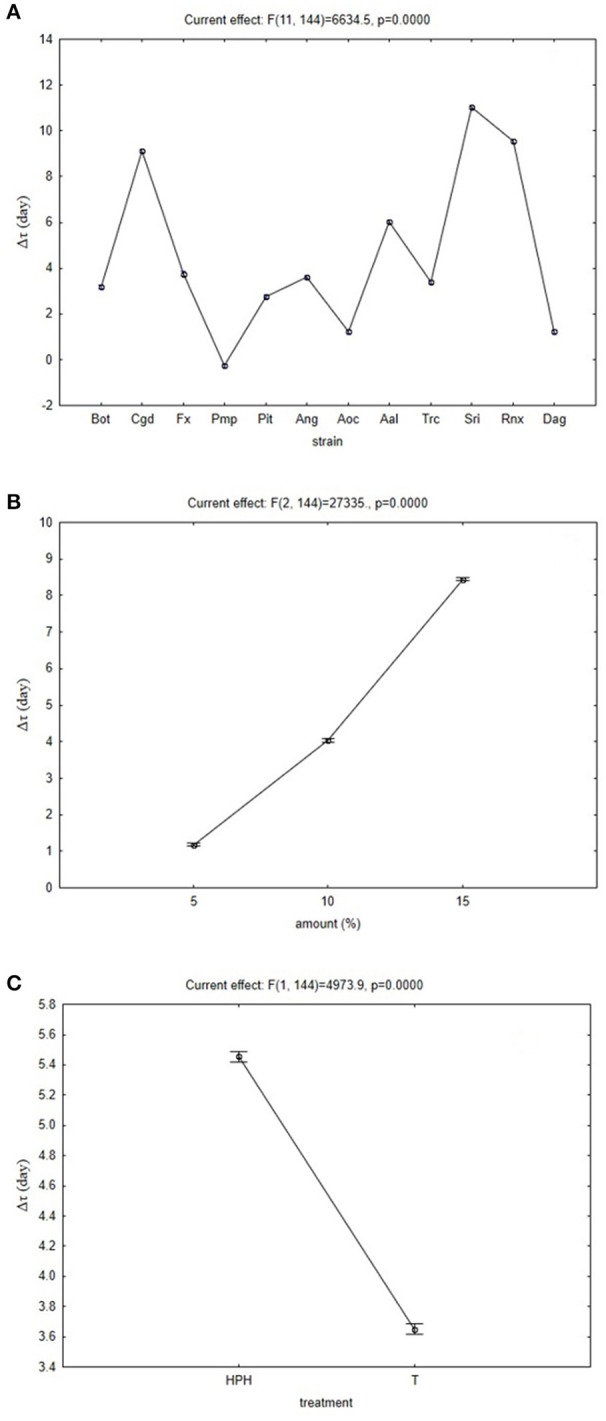
Effective hypothesis decomposition of the multifactorial ANOVA. Effects of fungi species (for numbers, see Table 1) **(A)**, and amount added **(B)** and treatment **(C)** for thermal-treated OMWW (T) and high-pressure homogenized OMWW (HPH). Vertical bars indicate 95% confidence intervals. The *F*-test and relative degrees of freedom are given at the top of each panel.

Another output of the multi-factorial approach is the decomposition of the trend of each factor, with this data shown in the hypothesis decomposition graphs (Figure 1). These show the quantitative effects of each independent factor. In terms of the fungal species (Figure 1A), some of these can be strongly inhibited by these OMWWs, such as *C. gloeosporioides, S. rolfsii* and *R. necatrix* (Figure 1A, Cgd, Sri, Rnx). The analysis defined their Δτ in the range from 8 to 10 days, thus indicating that their growth can be delayed by 8–10 days in comparison with their growth on PDA. Other fungi, such as *Pm. parasiticum* and *A. ochraceus* (Figure 1A, Pmp, Aoc), showed a lower Δτ, thus suggesting that their growth was not affected by these treated OMWWs.

Figure 1B shows the quantitative effects of the amount of treated OMWW added, where, as expected, increasing the amounts of TT-OMWW and HPH-OMWW in the minimal media resulted in a general delay of fungal growth. Finally, the effects of the high-temperature and HPH treatments might increase or decrease the effects of TT-OMWW and HPH-OMWW on the fungal growth (Figure 1C). Here, the HPH-OMWW promoted increased bioactivity of the OMWW, with increased mean Δτ from 3.0 days to 4.8 days. This relies only on the effects of the OMWW treatment, and is independent of the other two factors (i.e., fungal species, amount of treated OMWW added).

**Figure 2:**
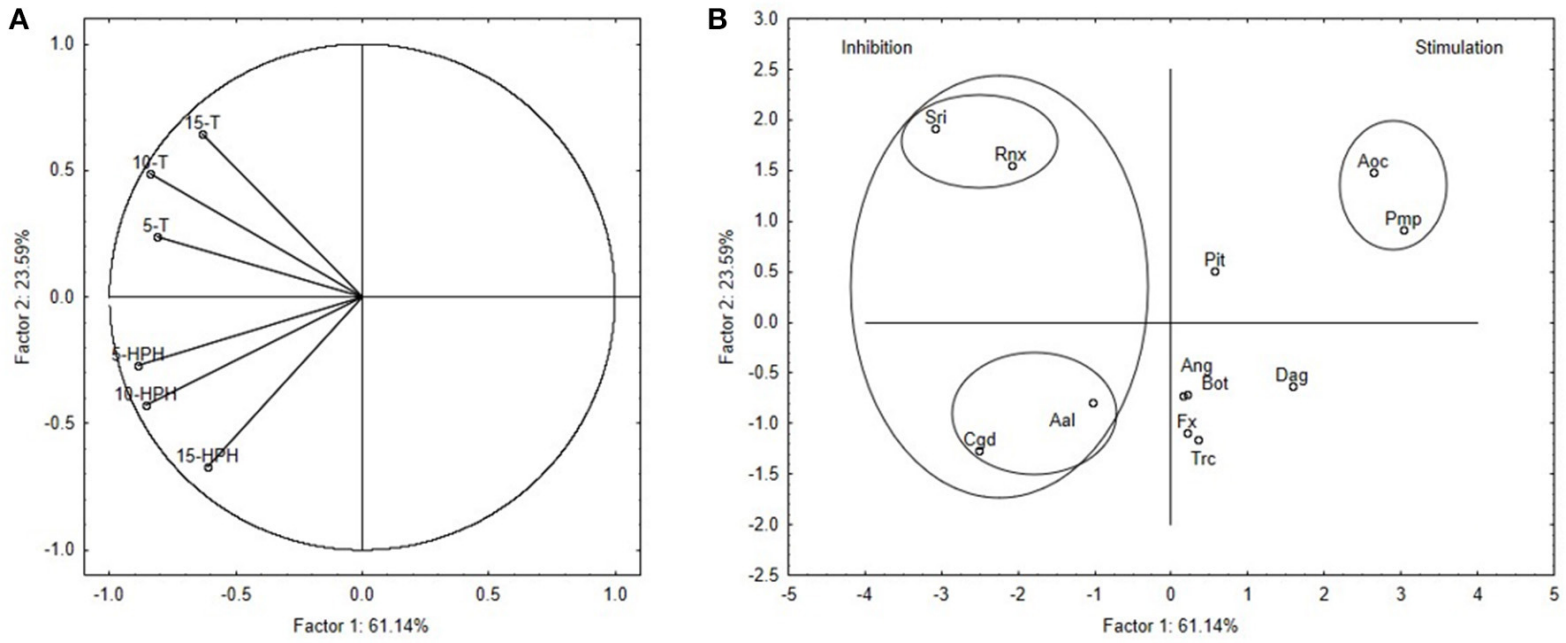
Principal Component Analysis run on Δτ values. **(A)** Projection of variables. Codes indicate percentage addition of thermal-treated OMWW (T) and high-pressure homogenized OMWW (HPH). **(B)** Projection of cases. For fungal species, see Table 1.

As a final step, a multivariate approach was carried out to group the different fungal species and to select the most suitable ones for further studies. Δτ from the media supplemented with TT-OMWW and HPH-OMWW were used as the input values for this Principal Component Analysis, with the outputs illustrated in Figure 2A (variable projection) and Figure 2B (case projection).

The PC1 and PC2 factors accounted for 84.73% of the total variability (Figure 2A). Here, variables 5-T, 10-T, 5-HPH, and 10-HPH (i.e., Δτ for the 5% and 10% TT-OMWW and HPH-OMWW media) were mostly related to PC1 (correlations, 0.814–0.885), while variables 15-T and 15-HPH showed similar correlations to both PC1 and PC2 (0.612–0.671).

This principal component analysis highlighted two groups for the fungal species (Figure 2B). The first was in quadrants II and III and comprised *C. gloeosporioides*, *A. alternata, S. rolfsii*, and *R. necatrix*, the growth of which was strongly inhibited by the addition of the OMWWs. This group is not homogeneous, because on the media containing 15% treated OMWWs, some differences were seen. Here, *S. rolfsii* and *R. necatrix* were more affected by 15% TT-OMWW, while *C. gloeosporioides* and *A. alternata* were more affected by 15% HPH-OMWW.

The second group of fungal species was in quadrant I, and included *A. ochraceus* and *Pm. parasiticum*. These two fungi were resistant to the treated OMWWs, and showed negative Δτ (i.e., stimulation of fungal growth by treated OMWWs). Finally, there is the remaining miscellaneous group that included the fungi with variable trends: *L. theobromae, F. oxysporum, P. italicum, A. niger, Trichoderma* sp., and *D. amygdali*.

As a last step, some preliminary chemical analyzed were done on OMWW. The control batch had a value of phenols of 11.25 ± 0.77 g L^−1^; this parameter increased to 16.34 ± 0.32 g L^−1^ in TT-OMWW, whereas it was at 10.99 g L^−1^ in HPH-OMWW. The Folin's method only assesses the total amount of phenols; however, this preliminary result could suggest a possible degradation of complex phenols because of the thermal treatment with an increase of the total phenolic compounds.

This process probably did not occur in HPH-OMWW, as the amount of phenols was not significantly different from the untreated batch. The content of proteins was at 373.05 ± 9.43 μg mL^−1^ and was not affected by the kind of treatment.

## Discussion

In recent years, several studies have evaluated OMWWs in different sectors, including agriculture, bioenergy production, bioconversion, and extraction of the valuable phytochemical compounds in OMWWs (Christodoulou et al., 2008; El Hajjouji et al., 2008; Ntaikou et al., 2009; Dermeche et al., 2013). To date, various physicochemical, biological, and combined technologies have also been applied to OMWW residues, to reduce their pollutant effects, including precipitation, coagulation, extraction, sedimentation, ion exchange, adsorption on active carbon, chemical and advanced oxidation, centrifugation and ultrafiltration, advanced oxidation processes and ultrafiltration, fungal pretreatment, and anerobic digestion and ultrafiltration (Jaouad et al., 2016). The most suitable treatment for these OMWWs and their residues on any large scale appears to be the conventional activated sludge process (Sipma et al., 2010). The activated sludge processes produce a large amount of excess sludge, which may cause a serious of environmental and health issues (Zhang et al., 2009; Luo et al., 2013). However, application of this kind of treatment to biodegradation of OMWWs might not be effective because the phenolic compounds present can inhibit the microbial metabolism of the sludge. Aerobic biological treatments using different microorganisms, including fungi (Dias et al., 2004) or bacteria (Knupp et al., 1996), are widely used and environmentally friendly.

The present study addresses the use of OMWW in agriculture to control fungal growth, with the second aim to determine the effects of two initial treatments on the bioactivities of the OMWW. Based on these data, the tested fungal species generally followed two trends. The first was for an increased τ for either the TT-OMWW or HPH-OMWW media. This indicates that these TT-OMWW and HPH-OMWW can have positive effects in the control of these fungal pathogens, through their polyphenols content.

HPH pretreatment can improve the sludge anaerobic digestion, above all if combined with an alkaline pH (Fang et al., 2014); pressure and number of cycles influence the effect of HPH, as they both improve sludge disintegration (Zhang et al., 2012). At high pressure and with repeated HPH-cycles, the disintegration degree and soluble chemical oxygen demand increased, thus resulting a release of organic matter (Fang et al., 2015).

Eight of the 12 fungi investigated here showed major slowing of their growth on HPH-OMWW: *L. theobromae, D. amygdali, C. gloeosporioides, A. alternata, F. oxysporum, Trichoderma* sp., *S. rolfsii*, and *R. necatrix*. These data are in agreement with Telles et al. (2017), who studied the profiles of phenolic compounds and their relationships with the defense mechanisms of bean against fungal contamination and the amylase inhibitory activity from fungi. They reported that the phenolic compounds could potentially inhibit fungal attack and/or prevent aflatoxin production in bean. Moreover, plant defense against fungal infection is influenced by different biotic and abiotic factors, such as the phenolic composition of specific plant tissues, which can prevent pathogen attacks (Mendes et al., 2013). The aromatic ring of phenols, along with –OH groups, play a major role in plant defense mechanisms (Richardson, 1980; Guajardo-Flores et al., 2013; Ramírez-Jiménez et al., 2014; Wang et al., 2015). Lambert et al. (2012) studied the antifungal role of phenols against grapevine wood decay against trunk disease fungi and found that hydroxystilbenoids and vitisin A and B significantly inhibited fungal growth, except for *Pm. aleophilum* and *Pm. parasiticum*.

Altintas et al. (2013) assessed the composition and the antibacterial and antifungal activities of Origanum essential oils. Thymol and carvacrol were the major components of this oil and exerted a significant effect on strawberry anthracnose-causing fungal plant pathogens *Colletotrichum acutatum, C. fragariae*, and *C. gloeosporioides* using the direct overlay bioautography assay. Thymol demonstrated antifungal activity and produced growth inhibition of *Phomopsis obscurans* (Ellis & Everh.) B. Sutton in experimental conditions, such as *D. amygdali* in our research. They suggested that fungi (and many other organisms) do not always respond to chemical agents in a concentration-dependent manner. Mean fungal growth of thymol and carvacrol tested separately and mixed together indicated that these phenolic compounds are not good natural fungicides, while the bioassay data indicated that thymol and carvacrol may act more as growth suppressive compounds in the plant and not as fungitoxic defense compounds against invading pathogens.

Other researchers have found that phenolic compounds can inhibit *Alternaria* spp. (Brenes et al., 2011); *F. oxysporum* (Patel and Saraf, 2017); *Trichoderma* sp. (Zheng et al., 2017); *R. necatrix* (Lee, 2000).

For the antifungal effects of OMWWs, the data from the present study are in line with the findings of Lops et al. (2008) for *S. rolfsii*, the causal fungal agent of collar rots as soil-borne pathogens of horticultural crops.

The second trend that was recorded in the present study was showed for *A. ochraceus* and *Pm. parasiticum*, which experienced decreased τ when grown on both TT-OMWW and HPH-OMWW at 5% and 10%. This defines a positive effect of the phenols on fungal growth, as previously reported by Bevilacqua et al. (2017). Martínková et al. (2016) reported that new promising wild-type producers have emerged of the enzymes for the degradation of phenols in model mixtures or real wastewaters and a number of recombinant strains were also constructed, based mainly on yeasts or *Aspergillus* strains as hosts, indeed a variety of processes were proposed at the lab scale for using these fungi and their phenol oxidases in the bioremediation.

Not ligninolytic *Aspergillus* species colonize soils and sediments contaminated with toxic chemicals. Thus, they could be goof tool for the bioremediation, because of their nonylphenol metabolism. The removal of nonylphenol by *A. versicolor* from waste and water pollutants can be used in bioreactors or on large-scale in sewage treatment plants where the fungus can be used as an inoculum for bioremediation processes (Krupinski et al., 2014).

In the same way, Sivasubramanian and Namasivayam (2015) developed a microbial consortium consisting of five phenol-degrading strains isolated from environmental source. The consortium could degrade 99% of 1,000 mg L^−1^ phenol after 72 h incubation with a biomass increase from 2.6 × 10^7^ to 4 × 10^12^ CFU mL^−1^. Characterization of the members revealed that consortium consisted of *Candida tropicalis* (1.2 × 10^5^ CFU mL^−1^), *Aspergillus fumigatus* (1.1 × 10^5^ CFU mL^−1^), *Candida albicans* (3.0 × 10^3^ CFU mL^−1^), *Candida haemulonis* (2.6 × 10^3^ CFU mL^−1^) and *Streptomyces alboflavus* (1.1 × 10^5^ CFU mL^−1^).

Another interesting finding in the present study was the different effects of the thermal treatment and HPH on the antifungal activities of these OMWWs. The statistical analysis suggests stronger bioactivity for HPH-OMWW, potentially due to preservation of the phenols through the treatment, as indicated by Varela-Santos et al. (2012).

The production of OMWW is seasonal, and in October to December, large volumes of OMWW are produced. These are strongly contaminated by a composite microbiota of bacteria, yeast, and fungi (McNamara et al., 2008), and storage of OMWW at room temperature or under refrigeration generally results in degradation of the phenolic compounds (Sinigaglia et al., 2010). Therefore, OMWW should be initially treated before storage, to assure longer use and bioactive potential. This study represents the first report on the evaluation of the effects of different stabilization approaches carried out for the OMWW raw material, where HPH appears to be a promising way forward. However, the results here and the effects of TT-OMWW on some of these fungi suggest that the effects of OMWW, as well as the influence of the initial treatments, also depend upon the fungal species. Indeed, some of these fungi were strongly affected by TT-OMWW, which thus suggests that the decomposition metabolites that originate from the thermal treatment might also have roles in the antifungal activity of TT-OMWW.

In conclusion, the disposal of OMWW represents a challenge for the environment, although it might also be an opportunity. This study addressed the use of low amounts of OMWWs, which can be used to counteract the effects of some soil-borne and/or pathogenic fungi in agriculture, including *L. theobromae, Pm. parasiticum, D. amygdali, C. gloeosporioides, A. alternata, F. oxysporum, Trichoderma* sp., *S. rolfsii*, and *R. necatrix*. In addition, two initial treatments of OMWW (temperature, HPH) were studied to determine whether they influence the bioactivities of the resulting OMWWs. These data suggest general improved benefits following HPH, as probable positive effects of the phenols, although there are some exceptions to this generalized statement.

## Author contributions

MC, MS, AC, and AB: conceived this study; FC, MR, DC, and CC: performed the analyses. AB and MC: performed the statistical analyses; FC and AB: wrote the paper; MC: funded the research; All authors edited and approved the final manuscript.

### Conflict of interest statement

The authors declare that the research was conducted in the absence of any commercial or financial relationships that could be construed as a potential conflict of interest.
